# Terrestrial Rabies and Human Postexposure Prophylaxis, New York, USA

**DOI:** 10.3201/eid1603.090298

**Published:** 2010-03

**Authors:** Millicent Eidson, Anissa K. Bingman

**Affiliations:** University at Albany School of Public Health, Rensselaer, New York, USA (M. Eidson, A. Bingman); New York State Department of Health, Albany, New York, USA (M. Eidson)

**Keywords:** rabies, lyssavirus, neurologic disorder, animals, New York, viruses, zoonoses, viruses, dispatch

## Abstract

During 1993–2002, cats accounted for 2.7% of rabid terrestrial animals in New York but for one third of human exposure incidents and treatments. Nonbite exposures and animals of undetermined rabies status accounted for 54% and 56%, respectively, of persons receiving rabies treatments.

Rabies has an almost 100% case-fatality rate and requires considerable resources for control (*1*). In the United States, canine rabies is controlled with vaccination and control of dogs (*2*). Infection occurs primarily from bite wounds. In US cases diagnosed before death, patients died 6–43 days after clinical onset (*3*). Although <10 human cases have been diagnosed annually since 1990 (*2*) in the United States, potential exposure incidents and rabies postexposure prophylaxis (PEP) of humans are not rare. PEP is the treatment regimen for 1 person, with 2–5 vaccine injections and immune globulin, depending on prior vaccination history. PEP is unnecessary if an animal is not rabid at exposure.

A rabies outbreak in raccoons in the mid-Atlantic states in 1977 (*4*) reached New York state, which has many areas with land types favored by raccoons (*5*,*6*), in 1990. In this study, we identified terrestrial rabies trends statewide in New York, with an aim toward prioritizing control. Previous analyses have focused on only part of the state (*7*) or on a shorter time period (*8*).

## The Study

In New York, need for PEP is determined by outcome of 10-day confinement (of all domestic animals) or laboratory testing (all species). Healthcare providers report suspected rabies exposures to local health departments, which absorb authorized PEP costs beyond those borne by third-party payers and partial reimbursement by the New York State Department of Health (*9*).

We analyzed exposure data collected electronically during 1993–2002. Exposures to bats and humans, animals submitted only for surveillance, and data from New York City (not part of the reporting system) were excluded. Rabies was diagnosed by direct fluorescent antibody staining. We analyzed data with SAS version 9.2 (SAS Institute, Cary, NC, USA) using US census data for rates (www.factfinder.census.gov). Because of skewed distributions, we used Spearman rank correlation coefficients for measures of association.

The number of terrestrial animals submitted declined 56% from 10,552 in 1993 to 4,631 in 2002. The number and proportion of rabid animals, which decreased from 2,637 (25.0%) in 1993 to 608 (13.1%) in 2002, were strongly associated with the number of submitted animals (Spearman r = 0.99, p<0.0001).

For 70.4% of the 13,004 exposure incidents during 1993–2002, an animal was not submitted for testing (Table 1). These incidents accounted for 10,097 (55.6%) of the 18,154 persons receiving PEP. Untestable and positive animals accounted for 2.6% and 23.4% of PEP, respectively. For 3.6% of exposure incidents, PEP began before rabies was ruled out.

**Table 1:** Terrestrial rabies–associated exposure incidents and rabies PEP use, by animal test result, New York, USA, 1993–2002*

Animal test result	No. (%) incidents	No. (%) PEP uses
Positive	3,047 (23.4)	7,032 (38.7)
Negative	469 (3.6)	551 (3.0)
Untestable	340 (2.6)	474 (2.6)
Not tested	9,148 (70.3)	10,097 (55.6)
Total	13,004 (100.0)	18,154 (100.0)

*Each rabies exposure situation in which >1 persons underwent PEP was defined as an incident. Excludes New York, NY. PEP, postexposure prophylaxis.

**Figure 1:**
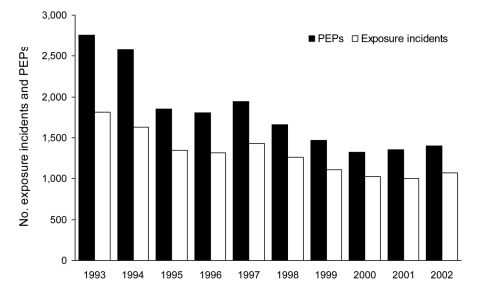
Terrestrial rabies–associated exposure incidents and postexposure prophylaxis (PEP) use, by year, New York (excluding New York City), USA, 1993–2002.

Exposure incidents declined 45%, from 1,815 in 1993 to 1,006 in 2001 (Figure 1). PEP decreased from 2,755 (25.3 PEPs/100,000 persons) in 1993 to 1,327 in 2000 (12.1 PEP/100,000 persons). Each year, the number of persons receiving PEP correlated with the number of submitted animals (Spearman r = 0.94, p<0.0001) and rabid animals (Spearman r = 0.95, p<0.0001). Although fewer cats (303) than raccoons (8,318) were rabid, cats accounted for the most exposure incidents (4,266 [32.8%]) and PEP (5,777 [31.8%]) (Table 2). Dogs accounted for 3,052 (23.5%) exposure incidents and 3,435 (18.9%) PEP. In New York, dogs and cats accounted for a high proportion of PEP from animals without rabies determination (85.3% and 67.6%, respectively). Raccoons accounted for 3,298 (25.4%) exposure incidents and for 5,210 (28.7%) PEP. From 1993 to 2002, the proportion of PEP attributed to raccoons changed from 48% to 22%; cats, from 21% to 35%; and dogs, from 11% to 22%.

**Table 2:** Terrestrial rabies–associated exposure incidents, number of rabid animals, and PEP use, by type of animal, New York, USA, 1993–2002*

Animal	No. exposure incidents	Total no. rabid animals	PEP use		
			Total no. uses	No. related to untested animals	No. related to nonbite incidentss†
Wild					
Raccoon	3,298	8,318	5,210	1,488	4,534
Fox	398	390	620	187	318
Skunk	637	1,894	987	302	839
Other	544	152	655	453	328
Domestic					
Dog	3,052	28	3,435	2,930	467
Cat	4,266	303	5,777	3,907	2,119
Other	187	143	668	63	625
Other/unknown	622	7	802	767	519
Total	13,004	11,235	18,154	10,097	9,749

*Each rabies exposure situation in which >1 persons underwent PEP was defined as an incident. Excludes New York, NY. PEP, postexposure prophylaxis. †Scratches, saliva/nervous system tissue exposure, mucous membrane exposure, indirect exposure, or unknown.

In 43 New York counties with populations <200,000, the PEP rate averaged 33.7/100,000 (range 8.4–81.3/100,000). The 14 larger counties (populations >200,000) had significantly lower PEP rates (9.8/100,000, range 0.5–21.8/100,000; p<0.0001) and PEP per exposure incident (p<0.0001) but accounted for 42.6% of PEP.

**Figure 2:**
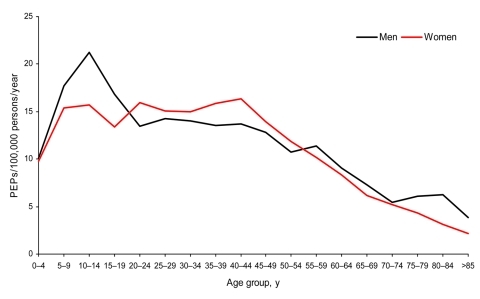
Rate of postexposure prophylaxis (PEP) use per 100,000 persons per year, by sex and 5-year age groups, New York (excluding New York City), 1998–2002.

During 1998–2002 when sex and age of exposed persons were reported, data were missing for 211 of 7,221 PEP reports. Persons who received PEP did not differ by sex (3,625 male, 3,569 female). PEP rates were highest for children 10–14 years of age (Figure 2). For male patients, PEP rates were lower in older age groups; for female patients, rates were highest in the 40–44-year group. Female patients received PEP significantly more often because of cat exposures than did male patients (1,736 vs. 1,053; p<0.0001). Male patients received PEP significantly more often from dog (984 vs. 583; p = 0.0005) and raccoon (767 vs. 595; p = 0.05) exposures than did female patients. For each age group, except the >85-year age group, female patients received PEP more often from cat exposures and male patients more often from dog exposures.

The 8,405 bites accounted for 46.3% of PEP. A total of 1,114 (6.1%) of PEP occurrences were associated with scratch exposures and 3,707 (20.4%) with saliva/nervous tissue exposures. For indirect or unknown types of contact, 4,298 (27.2%) PEP occurred. PEP for direct contact significantly exceeded that for indirect or unknown contact for the study period (p<0.0001) and for each year except 1993. Bites accounted for significantly more PEP because of dog and cat exposures (86.4% vs. 63.3%; p<0.0001) than did scratches or saliva/nervous tissue exposures. Raccoon exposures more frequently resulted from saliva/nervous tissue exposure than from bites (22.4% vs. 13.0%; p<0.0001). Most PEP resulting from indirect exposures (64.5%) was from raccoons.

Of 7,221 PEP occurrences during 1998–2002 when local health department authorization was reported, 6,846 (94.8%) were reported as authorized. PEP start date was reported for 6,786 (94.0%). Of 6,264 persons not reported as previously vaccinated, 5,574 (89.0%) received 5 vaccine doses and 5,563 (88.8%) received human rabies immune globulin. Of 522 persons previously vaccinated, 507 (97.1%) received 2 vaccine doses.

PEP completion was not reported (no report received) for 716 (11%) persons; 701 had no prior treatment history. Most (79%) incomplete PEP in New York was associated with animals not captured for rabies determination. Of 119 PEP associated with rabies-negative animals, 108 (91%) were not completed. PEP were not started for 17 (1%) and were not completed for 34 (2%) of the 2,217 PEP associated with rabid animals. Completion rates did not differ by patient sex. Most (697 [97%]) incomplete PEP was from direct contact exposures, primarily bites (87%). A total of 33 (9%) of 376 persons with adverse reactions did not complete treatment. Incomplete PEP was associated more often with exposures to dogs (42%) and cats (42%) than to other species.

The rate in New York was lower than that in Massachusetts when its epizootic was well established in 1995 (*10*), perhaps because New York requires treating physicians to consult with local public health authorities. Similar to rates in New York, PEP rates in Ontario, Canada, decreased as fox rabies became enzootic and were weakly but significantly associated with animal rabies (*11*). This association may be due to epizootic-related reductions in animal populations, resulting in fewer rabid animals and human contacts. Unlike New York, in Kentucky PEP occurred more frequently after exposures to dogs than cats (*12*). In Kentucky, the proportion of incomplete PEP was the same as in New York (Michael Auslander, pers. comm., 2008). Treatment completion rates for New York and Kentucky were higher than those in a study of 11 US emergency departments (65%) (*13*). In Florida, 22% of PEP were inappropriate according to a state algorithm (*14*); in New York, local health departments report few unauthorized PEP administrations.

## Conclusions

In New York, over time and with education, PEP associated with indirect exposures apparently can be reduced. Of most concern is the 55.6% of PEP associated with animals of undetermined rabies status. More efforts are needed to capture exposing animals to rule out both rabies and the need for PEP. Capturing exposing animals should be a major component of animal control efforts that along with vaccination have been successful at reducing rabies risks.
